# Synthesis, Characterization, and In Vitro Insulin-Mimetic Activity Evaluation of Valine Schiff Base Coordination Compounds of Oxidovanadium(V)

**DOI:** 10.3390/biomedicines9050562

**Published:** 2021-05-17

**Authors:** Mihaela Turtoi, Maria Anghelache, Andrei A. Patrascu, Catalin Maxim, Ileana Manduteanu, Manuela Calin, Delia-Laura Popescu

**Affiliations:** 1 Medical and Pharmaceutical Bionanotechnologies Laboratory, Institute of Cellular Biology and Pathology Nicolae Simionescu of the Romanian Academy, 8 B.P. Hasdeu, 050568-Bucharest, Romania; maria.anghelache@icbp.ro (M.A.); ileana.manduteanu@icbp.ro (I.M.); 2 Department of Inorganic Chemistry, Faculty of Chemistry, University of Bucharest, 23 Dumbrava Roşie, 020464-Bucharest, Romania; andrei_alunel@yahoo.com (A.A.P.); catalin.maxim@chimie.unibuc.ro (C.M.)

**Keywords:** oxidovanadium(V) coordination compounds, L-/D-valine Schiff base ligands, crystallography: insulin-mimetic compounds, anti-diabetic activity

## Abstract

Type 2 diabetes became an alarming global health issue since the existing drugs do not prevent its progression. Herein, we aimed to synthesize and characterize a family of oxidovanadium(V) complexes with Schiff base ligands derived from L-/D-valine (val) and salicylaldehyde (sal) or *o*-vanillin (van) as insulin-mimetic agents and to assess their potential anti-diabetic properties. Two new oxidovanadium(V) complexes, [{V^V^O(R-salval)(H_2_O)}(μ_2_-O){V^V^O(R-salval)}] and [{V^V^O(R-vanval)(CH_3_OH)}_2_(μ_2_-O)], and their S-enantiomers were synthesized and characterized. The compounds exhibit optical activity as shown by crystallographic and spectroscopic data. The stability, the capacity to bind bovine serum albumin (BSA), the cytotoxicity against human hepatoma cell line, as well as the potential anti-diabetic activity of the four compounds are investigated. The synthesized compounds are stable for up to three hours in physiological conditions and exhibit a high capacity of binding to BSA. Furthermore, the synthesized compounds display cytocompatibility at biologically relevant concentrations, exert anti-diabetic potential and insulin-mimetic activities by inhibiting the α-amylase and protein tyrosine phosphatase activity, and a long-term increase of insulin receptor phosphorylation compared to the insulin hormone. Thus, the in vitro anti-diabetic potential and insulin-mimetic properties of the newly synthesized oxidovanadium(V) compounds, correlated with their cytocompatibility, make them promising candidates for further investigation as anti-diabetic drugs.

## 1. Introduction

The prevalence of type 2 diabetes has increased alarmingly in recent years, therefore the development of new effective treatment options to increase insulin sensitivity is a challenge for many researchers [1]. Type 2 diabetes is characterized by high blood glucose levels as a consequence of defects in the insulin signaling pathway, including the decrease in the phosphorylation of insulin-stimulated receptor tyrosine kinase (INS R) [2]. Although there are numerous hypoglycemic drugs known, such as insulin analogs, metformin, sulfonylureas, thiazolidinediones, sodium-glucose co-transporter-2 inhibitors, or acarbose, the diabetic disease progresses over time [3]. The development of new drugs to mimic the biological effects of insulin is therefore essential.

Therapeutic applications of vanadium and its compounds have been extensively studied since the 1990s [4]. Hence, a large number of in vitro and in vivo studies have demonstrated that vanadium salts and their well-known complexes have beneficial properties in diseases such as diabetes [4,5,6,7] and cancer [8,9]. Various complexes of oxidovanadium(IV/V) have been synthesized in the past three decades starting from the vanadyl ion and different ligands, such as amino acids, peptides, and Schiff bases, to improve compound stability, solubility, and bioavailability, properties mandatory for their biological effects [8,10,11,12,13].

Albumin is the most important protein in the blood involved in drug transport to various tissues. The capacity of vanadium complexes to bind to bovine serum albumin (BSA), as well as anti-diabetic properties of many synthesized vanadium complexes were previously reported [5,6,12,14,15,16,17].

Hyperglycemia and an imbalance in glucose homeostasis occur in diabetes. Salivary and pancreatic α-amylases initiate glucose metabolism by hydrolyzing the food carbohydrates in the digestive system. Recently, the ability of oxidovanadium(IV) complexes with L-amino acids to inhibit the pancreatic α-amylase activity was showed in vitro [18]. The anti-diabetic activity of vanadium complexes was associated with their capacity to mimic the effects of the insulin hormone and to activate the insulin signaling pathway, which is essential for lowering the blood glucose levels [2].

Intracellular protein tyrosine phosphatases (PTPs) dephosphorylate the phosphor-tyrosine residues of some proteins. Particularly, PTP1B is involved in the regulation of insulin signaling in the absence of insulin stimulation and its specific inhibition turned out to be essential for treating diabetes [19]. The involvement of vanadium compounds in the inhibition of enzymatic PTP activity has already been established [2,14,20,21]. Recent X-ray diffraction studies have shown that the mechanism underlying the inhibition of PTPs by vanadium compounds is the interaction between vanadium and the active site of most phosphatases, a process that may involve dissociation of the initial complex [14,22].

This study aims to synthesize and characterize a family of oxidovanadium(V) complexes with Schiff base ligands derived from L-/D-valine and salicylaldehyde or *o*-vanillin as insulin-mimetic agents and to assess their potential anti-diabetic properties.

Herein we developed and physico-chemically characterized two new oxidovanadium(V) complexes with Schiff base ligands derived from L-/D-valine and salicylaldehyde or *o*-vanillin and their S-enantiomers. We also report the in vitro assessment of therapeutic behavior of the four vanadium-based compounds, which are stable in PBS at physiological pH and temperature for up to three hours and display cytocompatibility at biologically relevant concentrations. The compounds have a high capacity to bind serum albumin, exhibit the ability to inhibit the activities of α-amylase and intracellular PTPs, and furthermore, improve the phosphorylation of the insulin receptor in the human hepatoma (HepG2) cell line. Our data may provide the basis for further investigations of these vanadium-based compounds as drugs in diabetes therapy.

## 2. Materials and Methods

### 2.1. Materials

The reagents used in this study were analytically pure and were purchased from commercial manufacturers. α-Amylase from porcine pancreas, bovine serum albumin (BSA), 3, 5-dinitrosalicylic acid (DNS), D-/L-valine, dimethyl sulfoxide (DMSO), Dulbecco’s modified Eagle’s medium (DMEM), 4-(2-hydroxyethyl)piperazine-1-ethanesulfonic acid (HEPES), ethylenediaminetetraacetic acid (EDTA) tetrasodium salt dihydrate, glycerol, 2-mercaptoethanol, salicylaldehyde, sodium orthovanadate (Na_3_V^V^O_4_), sodium chloride, sodium hydroxide, sodium dodecyl sulfate (SDS), p-Nitrophenyl Phosphate (pNPP), starch, o-vanillin, Tris, V^IV^OSO_4_•3H_2_O were from SIGMA-Aldrich (Merck KGaA, Darmstadt, Germany). Acarbose, bromophenol blue, fetal bovine serum, human recombinant insulin, penicillin, streptomycin, 2, 3-Bis-(2-methoxy-4-nitro-5-sulfophenyl)-2H-tetrazolium-5-carboxanilide (XTT), and phenazine methosulfate were from Thermo Fisher Scientific (Waltham, MA, USA). Cisplatin was from Tocris Bioscience (Minneapolis, MN, USA). The 96-well UV/ Vis micro test plates and cell culture dishes were from Corning (New York, NY, USA)/ Ratiolab GmbH (Dreieich, Germany) and TPP^®^ (Trasadingen, Switzerland).

### 2.2. Physical Measurements and Elemental Analysis

Elemental analysis was used for experimental determination of percentage content of the carbon, hydrogen, and nitrogen elements in each vanadium compound using EuroEA Elemental Analyser (software Callidus™) system.

The IR spectra were recorded in KBr pellets, at room temperature, within 4000 ÷ 400 cm^−1^, using the FT-IR-Bruker Tensor-V-37 spectrometer. Data processing was done with OPUS program.

The UV-Vis absorption spectra were recorded using the Jasco V580 spectrophotometer, in the 500 ÷ 190 nm or 900 ÷ 190 nm range, in 10 × 10 mm quartz cells. The oxidovanadium(V) complexes, salicylaldehyde, and *o*-vanillin were solubilized in DMSO, while D-/L-valine and V^IV^OSO_4_•3H_2_O in distilled water to form a 2 × 10^−1^ M stock solution. The working samples were prepared in phosphate-buffered saline (PBS) (1.36 × 10^−1^ M NaCl, 2.6 × 10^−3^ M KCl, 1.01 × 10^−2^ M Na_2_HPO_4_, 1.76 × 10^−3^ M KH_2_PO_4_, pH 7.4). The electronic spectra were measured for 1 × 10^−4^ M vanadium complexes, 5 × 10^−4^ M salicylaldehyde, and *o*-vanillin against DMSO—PBS and for 2 × 10^−3^ M amino acid and 7.2 × 10^−2^ M V^IV^OSO_4_•3H_2_O against PBS. The final spectra were the average of three recordings. All measurements were done in triplicates. Spectra Manager Software package was used for data analysis.

The circular dichroism (CD) spectra of 5 × 10^−4^ M vanadium complexes were run on a Jasco J-1500 spectrophotometer equipped with Spectra Manager software, within 500 ÷ 200 nm, and were recorded against 0.25% DMSO-PBS instantly (T 0 h depicted) and at 9 h (T 9 h) after preparation of the working solutions (preserved at 37 °C). The final spectra are the average of nine recordings. The CD spectra for 2 × 10^−2^ M L-/D-valine were recorded against PBS. All measurements were done in triplicates.

Crystal structure determination and refinement. X-ray diffraction measurements for compounds [{V^V^O(R-salval)(H_2_O)}(μ_2_-O){V^V^O(R-salval)}] (**1a**) and [{V^V^O(R-vanval)(CH_3_OH)}_2_(μ_2_-O)] (**2a**) were performed on a STOE IPDS II diffractometer operating with Mo-Kα(λ = 0.71073 Å) X-ray tube with graphite monochromator. The structures were solved by direct methods and refined by full-matrix least-squares techniques based on F^2^. The non-H atoms were refined with anisotropic displacement parameters. Calculations were performed using the SHELX-2013 crystallographic software package. The structures were solved by direct methods using the SHELXS structure solution program. The H atoms attached to carbon were introduced in idealized positions using the riding model. A summary of the crystallographic data and the structure refinement for crystals **1a** and **2a** are given in Supplementary Table S3. CCDC reference numbers: 2070140-2070141.

### 2.3. Synthesis of the Oxidovanadium(V) Complexes

#### 2.3.1. Synthesis of [{V^V^O(R-salval)(H_2_O)}(μ_2_-O){V^V^O(R-salval)}] (**1a**) and [{V^V^O(S-salval)(H_2_O)}(μ_2_-O){V^V^O(S-salval)}] (**1b**)

The compound **1b** was previously reported by Cavaco et al. [13]. Both **1a** and **1b** were synthesized in this study following a slightly modified procedure. D-valine/L-valine (0.175 g, 1.5 mmol) and NaOH (0.08 g, 2 mmol) were dissolved in 30 mL of CH_3_OH. The mixture was added dropwise over a methanolic solution (5 mL) of salicylaldehyde (0.122 g, 1 mmol, 1.146 g/cm^3^) with the spontaneous occurrence of a yellowish solution. After 1 h at 323 K, an aqueous solution (2 mL) of VOSO_4_•3H_2_O (0.217 g, 1 mmol) was added dropwise and the magnetic stirring continued at 323 K for 1 h. The resulting mixture was filtered and the brown solution was left for 10 days. The dark green rhinestone crystals were formed by slow solvent evaporation.

[{V^V^O(R-salval)(H_2_O)}(μ_2_-O){V^V^O(R-salval)}]—Yield: 0.69 g (56.93%); Anal. Calc (%): C, 47.49; H, 4.61; N, 4.61. Found (%): C, 47.46; H, 4.39; N, 4.88. IR (KBr, pellets, cm^−1^): 451 ν(V-O), 571 ν(V-N), 761 ν_as_(V-O-V), 991 ν(V = O), 1286 ν(C_Ph_-O), 1446 ν_s_(COO^−^), 1554 ν(C = C), 1602 ν_as_(COO^−^), 1712 ν(C = N), 2966-2870 ν(C-H) methyl, 3701 ν(O-H). UV-Vis (λ_max_, ε, M^−1^*cm^−1^ for 1 × 10^−4^ M compound) in PBS, pH 7.4: 232 nm (35796), 274 nm (24957), 371 nm (5432).

[{V^V^O(S-salval)(H_2_O)}(μ_2_-O){V^V^O(S-salval)}]—Yield: 0.60 g (49.5%); Anal. Calc (%): C, 47.49; H, 4.61; N, 4.61. Found (%): C, 47.46; H, 4.36; N, 4.72. IR (KBr, pellets, cm^−1^): 451 ν (V-O), 571 ν(V-N), 761 ν_as_(V-O-V), 991 ν(V = O), 1286 ν(C_Ph_-O), 1446 ν_s_(COO^−^), 1554 ν(C = C), 1602 ν_as_(COO^−^), 1712 ν(C = N), 2966-2870 ν(C-H) methyl, 3701 ν(O-H). UV-Vis (λ_max_, ε, M^−1^*cm^−1^ for 1 × 10^−4^ M compound) in PBS, pH 7.4: 232 nm (36574), 274 nm (24733), 371 nm (5384).

#### 2.3.2. Synthesis of [{V^V^O(R-vanval)(CH_3_OH)}_2_(μ_2_-O)] (**2a**) and [{V^V^O(S-vanval)(CH_3_OH)}_2_(μ_2_-O)] (**2b**)

**2b** was synthesized for the first time by Guo et al. [12]. Both complexes were obtained as previously described [12].

[{V^V^O(R-vanval)(CH_3_OH)}_2_(μ_2_-O)] Yield: 0.40 g (56.33%); Anal. Calc for (%): C, 47.29; H, 4.78; N, 3.94. Found (%): C, 47.69; H, 4.42; N, 3.87. IR (KBr, pellets, cm^−1^): 459 ν(V-O), 597 ν(V-N), 744 ν_as_(V-O-V), 974 ν(V = O), 1259 ν(C_Ph_-O), 1411 ν_s_(COO^−^), 1570 ν(C = C), 1627 ν_as_(COO^−^), 1680 ν(C = N), 2966-2872 ν(C-H) methyl, 3702-3400 ν(O-H). UV-Vis (λ_max_, ε, M^−1^*cm^−1^ for 1 × 10^−4^ M compound) in PBS, pH 7.4: 233 nm (34090), 287 nm (20811), 391 nm (4586).

[{V^V^O(S-vanval)(CH_3_OH)}_2_(μ_2_-O)]—Yield: 0.42 g (59.15%); Anal. Calc (%): C, 47.29; H, 4.78; N, 3.94. Found (%): C, 47.65; H, 4.77; N, 3.75. IR (KBr, pellets, cm^−1^): 459 ν (V-O), 597 ν_s_(V-N), 744 ν_as_(V-O-V), 974 ν(V = O), 1259 ν(C_Ph_-O), 1411 ν_s_(COO^−^), 1570 ν(C = C), 1627 ν_as_(COO^−^), 1680 ν(C = N), 2966-2872 ν(C-H) methyl, 3702-3400 ν(O-H). UV-Vis (λ_max_, ε, M^−1^*cm^−1^ for 1 × 10^−4^ M compound) in PBS, pH 7.4: 233 nm (30692), 287 nm (20355), 391 nm (4659).

**Figure 1:**
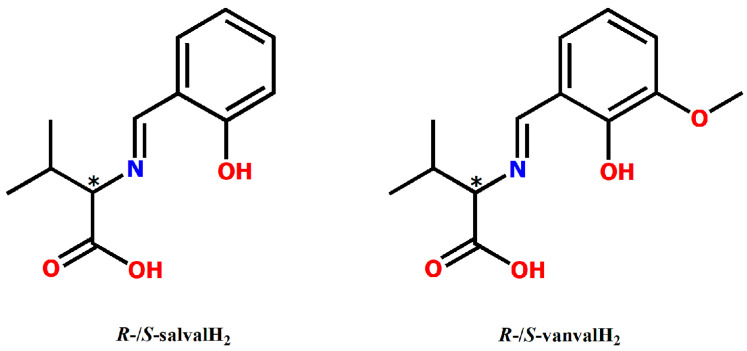
Structure formulas of the Schiff base ligands, *R*-/*S*-salvalH_2_ (salval = N-salicylidenvaline) and *R*-/*S*-vanvalH_2_ (vanval = 3-Methoxy-N-salicylidenvaline).

The structural formulas of the Schiff base ligands used in the synthesis of the four oxidovanadium(V) complexes are depicted in Figure 1.

### 2.4. Evaluation of the Solution Stability Over Time

Electronic absorption spectra for the 2 × 10^−4^ M solution of the four oxidovanadium(V) complexes in PBS at pH 7.4 were recorded outright on the prepared solutions (designated as T 0 h in the histograms), after the preparation at 1 h (T 1 h), 3 h (T 3 h), 6 h (T 6 h), 9 h (T 9 h), and 24 h (T 24 h), respectively. The prepared solutions were incubated at 37 °C, and the readings were performed over the entire 500 ÷ 230 nm range using a microplate reader spectrophotometer TECAN Infinite M200Pro and a 96-well UV micro test plate. The data were expressed as the average ± SD of three independent measurements.

### 2.5. In Vitro Biological Investigations

#### 2.5.1. Fluorescence Quenching of Serum Albumin

The capacity of the oxidovanadium(V) compounds to bind to bovine serum albumin (BSA) was monitored by fluorescence quenching assay [12]. A solution of 2 × 10^−6^ M BSA in PBS, pH 7.4, was incubated for 2 h at 37 b0C with each vanadium-based compound or V^IV^OSO_4_•3H_2_O at increasing molar concentrations (1 × 10^−6^ ÷ 2.5 × 10^−5^ M) or with 0.025% DMSO (negative control). The samples were excited at 295 nm and the emission spectra of all BSA solutions were recorded in the 300 ÷ 450 nm range using a JASCO FP-750 spectrofluorometer. The final spectrum was the average of three recordings measured at a scanning speed of 250 nm/minute, with an excitation and emission bandwidth of 5 nm. The maximum fluorescence intensity of BSA in PBS was recorded at 347 nm and was used as a benchmark for demonstrating the ability of the compounds to quench BSA fluorescence. In addition, by applying the Stern–Volmer equation [23], the mechanism by which the complexes bind to BSA was investigated as it is described in the Supplementary material.

#### 2.5.2. Inhibition of the α-Amylase Activity

The capacity of the obtained complexes to inhibit pancreatic α-amylase activity in vitro was analyzed using a slightly modified procedure of the one described by Apostolidis and Lee [24]. Thus, 100 µL solution of complexes (1.8 ÷ 2.5 × 10^−2^ M), 2.5 × 10^−2^ M V^IV^OSO_4_•3H_2_O, 2.5 × 10^−2^ M acarbose (positive control) and 12.5% DMSO (negative control) in reaction buffer (2 × 10^−2^ M sodium phosphate, pH 6.9 with 6 × 10^−3^ M sodium chloride) were pre-exposed to 100 µL of 20 units/mL α-amylase in reaction buffer for 10 min at room temperature. Then, 100 µL of 1% starch solution in reaction buffer were added and the samples were incubated at room temperature for 10 min. The reaction was stopped with 200 µL of 3, 5-dinitrosalicylic acid (DNS). The samples were heated at 90 ÷ 95 °C for 10 min and then left to cool down at room temperature. The reaction mixture was further diluted with 4 mL of distilled water. As a control for the inhibitory effect of acarbose, a sample containing starch and enzyme was used. The yellow/orange color intensity was measured using the microplate reader TECAN Infinite M200Pro at 540 nm. The experiment was performed in triplicate. The blank (sample without starch and enzyme) absorbance was subtracted from the absorbance of all samples and the activity of α-amylase was expressed as % of DMSO (negative control).

#### 2.5.3. Cell Culture

HepG2 cell line was purchased from American Type Culture Collection. The cells were grown in Dulbecco’s Modified Eagle’s Medium (DMEM) with 4.5‰ glucose, supplemented with 10% (*v*/*v*) fetal bovine serum, 100 units/mL penicillin, and 100 µg/mL streptomycin (complete medium). HepG2 cells were maintained at 37 °C in a 5% carbon dioxide incubator and were periodically sub-cultivated.

#### 2.5.4. Evaluation of Oxidovanadium(V) Complexes Cytotoxicity

To determine the cytotoxic concentration of the synthesized oxidovanadium(V) complexes and V^IV^OSO_4_•3H_2_O, HepG2 cells were incubated with increasing molar concentrations (1 × 10^−5^ ÷ 3 × 10^−4^ M) of compounds. Stock solutions of the compounds were prepared as described in the Physical measurements section. The stock solution of the positive control, cisplatin (3 × 10^−2^ M), was prepared in DMSO, and working concentrations between 2 × 10^−6^ and 3 × 10^−5^ M were used.

HepG2 cells (1.5 × 10^4^ cells/mL) were seeded in complete medium on a 96-well cell culture plate and were incubated at 37 b0C, as previously described. Upon reaching the confluence, the cells were exposed for 24 h to increasing molar concentrations of oxidovanadium(V) complexes, V^IV^OSO_4_•3H_2_O, and cisplatin prepared from stock solutions directly in the cell culture medium. Control cells were represented by cells exposed to free-complete medium and medium supplemented with 0.15% DMSO. The cytotoxicity of tested compounds on the HepG2 cell line was assessed by the 2, 3-bis-(2-methoxy-4-nitro-5-sulfophenyl)-2H-tetrazolium-5-carboxanilide) (XTT) cell viability assay. The method is based on the reduction of XTT, in the presence of phenazine methosulfate, to a water-soluble orange formazan derivative. The absorbance of the sample, due to the intensity of the orange color, which is proportional to the number of viable cells, was recorded at 450 nm using the Tecan Infinite M200Pro spectrophotometer. Cell viability was expressed as % of cells incubated with 0.15% DMSO (100% viability) and the results as the average ± standard deviation (SD) of three individual experiments, each performed in triplicate.

#### 2.5.5. Determination of Intracellular Total PTP Activity

HepG2 cells (1.0 × 10^5^ cells/mL) were seeded in complete medium on a 24-well cell culture plate and were incubated at 37 °C for 48 h. The cells were exposed for 3 h and 24 h to 2.5 × 10^−5^ M oxidovanadium(V) complexes, V^IV^OSO_4_•3H_2_O, and Na_3_V^V^O_4_. Control cells were represented by cells exposed to free-complete medium (negative control for treatment with V^IV^OSO_4_•3H_2_O and Na_3_V^V^O_4_) and medium supplemented with 0.0125% DMSO (negative control for treatment with complexes). The controls and treated cells were washed twice with PBS, lysed in 50 µL radio immune-protein assay buffer (RIPA), sonicated for 1 min, and centrifuged at 10,000× *g*, 4 °C, for 10 min [25]. PTP activity was determined using a slightly modified method [26]. Briefly, 10 µL of cell lysate was incubated with 90 µL of 10^−2^ M p-Nitrophenyl Phosphate (pNPP) in phosphatase buffer (10^−1^ M HEPES, 10^−3^ M EDTA tetrasodium salt, 10^−1^ M NaCl, pH 7.5) or 10^−2^ M p-Nitrophenyl Phosphate (pNPP) in phosphatase buffer supplemented with 10^−3^ M Na_3_VO_4_ (inhibitor of phosphatases) [27] for 30 min at 37 °C. The reaction was blocked with 1 M NaOH, and absorbance was measured at 405 nm with a Tecan Infinite M200Pro spectrophotometer.

The enzyme activity (EA) was calculated based on the formula: EA = [µmoles/min × µg] = 100 [vol] × ((A _(sample_ _− inhibitor)_-A _(sample + inhibitor)_)—(A _(blank_ _− inhibitor)_-A _(blank + inhibitor)_)) × 1/time [min] × total protein [µg] ×1/18,000 [molar extinction coefficient]. Total protein concentration (µg/mL) in cell lysate was determined by the BCA method using BSA standard [28]. The results were expressed as % of cells incubated with DMSO for each experimental time point. The data were expressed as the average ± SD of three individual experiments, each performed in duplicate.

#### 2.5.6. Quantification of the Phosphorylated form of Insulin Receptor

To investigate the effect of oxidovanadium(V) complexes on insulin receptor (INS R) phosphorylation, HepG2 cells (1.0 × 10^5^ cells/mL) were treated with 2.5 × 10^−5^ M oxidovanadium(V) compounds, V^IV^OSO_4_•3H_2_O, and 10^−7^ M insulin (positive control) for 3 and 24 h, respectively. As negative controls, cells exposed to free-complete medium (negative control for treatment with V^IV^OSO_4_•3H_2_O and insulin) and cells exposed to 0.0125% DMSO (negative control for treatment with complexes) were used.

After cell lysis, 50 µg of total protein was denatured by adding 1/5th volume of Laemmli’s buffer 6× (15% SDS, 0.01% bromophenol blue, 74% glycerol, 7.5 × 10^−2^ M Tris, pH 6.8, 1/8th volume of 2-mercaptoethanol) followed by heating at 95 °C for 5 min. The samples were loaded on a 5% SDS-PAGE gel and separated on a 10% SDS-PAGE gel electrophoresis (Mini-PROTEAN Tetra Cell, Bio-Rad Laboratories, Irvine, CA, USA).

The separated proteins were transferred (using a Trans-Blot Semi-Dry transfer cell, Bio-Rad) to a nitrocellulose membrane (0.45 µm, Bio-Rad), blocked with 1% BSA for 1 hour [29], and incubated overnight with the primary antibody: Mouse anti-insulin receptor-β chain (INS R-β) (1:500, Santa Cruz Biotechnology cat. No. sc-57342), rabbit anti-phosphorylated INS R-Y1162/3 (pINS R, 1:400, R&D Systems, Minneapolis, MN, USA cat. No. AF2507) and mouse anti-β-actin (1/2000, Bio-Rad cat. no. MCA5775GA). After washing, the membrane was exposed for 1 h to horseradish peroxidase-conjugated secondary antibodies (1:10,000, goat anti-rabbit IgG and goat anti-mouse IgG, Thermo Fisher Scientific cat. no. 32460 and 32430, respectively). To determine the target proteins, chemiluminescent detection (luminol-based enhanced chemiluminescence horseradish peroxidase substrate, Thermo Fisher Scientific) was performed with the G: Box Chemi XX6 System analyzer (Labgene Scientific, Châtel Saint-Denis, Switzerland). The pINS R protein expression was estimated relative to total INS R-β. β-actin was used as a reference protein. The data were expressed as the mean ± SD of two individual experiments performed in duplicate.

### 2.6. Statistical Analysis

Statistical evaluation was carried out by unpaired two-tailed Student’s *t*-test using GraphPad Prism 8 software. The results were expressed as mean ± standard deviation (SD) and *p* < 0.05 was considered to be statistically significant.

## 3. Results and Discussion

### 3.1. The IR Spectra of the Oxidovanadium(V) Complexes

The main infrared absorption frequencies (cm^−1^) for oxidovanadium(V) complexes and their precursors are depicted in Supplementary Tables S1 and S2, respectively.

The **1b** [13] and **2b** [12] structures and IR data have been previously elucidated, but given the technical and instrumental variability, we recorded the spectra for these two compounds as reference for the R-enantiomers, which had not been synthesized so far.

The appropriate superposition of IR spectra recorded for the two pairs of isomers, **1a**/**1b**, and **2a**/**2b**, reveals the enantiomeric relationship between them (Table S1), also confirmed by crystallographic data (see below). The IR spectra of **1a** and **1b** exhibit a low-medium absorption band between 3067 ÷ 3031 cm^−1^ that may be assigned to ν_s_(O-H) stretching vibration, evidencing the coordination of the O atom from water to vanadium centers [13]. In addition, a medium intensity band between 3702–3400 cm^−1^ was observed for **2a** and **2b** corresponding to the ν_s_(O-H) stretching frequency as a result of the coordination of the O atom from methanol to the vanadium atoms [12]. The low-medium intensity bands at 2966, 2936, and 2870 cm^−1^ for **1a**/**1b**, and at 2966, 2933, and 2872 cm^−1^ for **2a**/**2b** can be attributed to the methyl C-H stretching frequency. Furthermore, we noticed the absence of the bands in the 2625 ÷ 2110 cm^−1^ region associated with the amino acid NH bond observed for free valine (Supplementary Tables S1 and S2) [11]. Moreover, the presence of a very intense band at 1712 ÷ 1683 cm^−1^ for **1a**/**1b** and at 1680 cm^−1^ for **2a**/**2b** can be attributed to stretching frequencies of ν(C = N) from the vanadium-coordinated Schiff base ligands [12,13]. The asymmetric stretching vibrations of carboxylate, ν_as_(COO^−^) corresponds to the high-intensity band with a frequency of 1602 cm^−1^ for **1a**/**1b** and 1627 cm^−1^ for **2a**/**2b**. The medium-high intensity band with a frequency of 1394 cm^−1^ for **1a**/**1b** and 1411 cm^−1^ for **2a**/**2b** (Supplementary Table S1) can be assigned to the symmetric stretching vibrations of carboxylate, ν_s_(COO^−^) [12,30]. The vibration frequency (Δν) which represents the difference between the ν_as_(COO^−^) and the ν_s_(COO^−^) is greater than 200 cm^−1^ for **1a**/**1b** and **2a**/**2b**, highlighting the coordination of the deprotonated carboxyl groups, which belongs to the Schiff base, to vanadium atom in a monodentate manner [12,31]. The coordination manner of the carboxylate groups in the vanadium compounds is also supported by single-crystal X-ray analysis (see below). A medium to a high-intensity band at 1286 cm^−1^ for **1a**/**1b** and 1259 cm^−1^ for **2a**/**2b** can be assigned to the vibration of phenolic C-O bond (ν_s_C_Ph_-O) [31]. The presence of a moderate intensity band at 991 cm^−1^ for **1a**/**1b** and 974 cm^−1^ for **2a**/**2b** can be attributed to the V = O stretching vibration [12]. The intense band at 761 cm^−1^ for **1a**/**1b** and 744 cm^−1^ for **2a**/**2b** can be assigned to the asymmetric vibration stretching of V-O-V (ν_as_(V-O-V)) [12,32]. Strong to moderate intensity bands at 571 cm^−1^ and 451 cm^−1^ for **1a**/**1b** and 597–498 cm^−1^ and 459 cm^−1^ for **2a**/**2b** can be attributed to ν_s_(V-N) and ν(V-O) (carboxyl), respectively [12].

### 3.2. The Electronic and CD Spectra of Oxidovanadium(V) Complex Solutions

Electronic spectral data of obtained vanadium-based compounds are summarized in Table 1.

**Figure 2:**
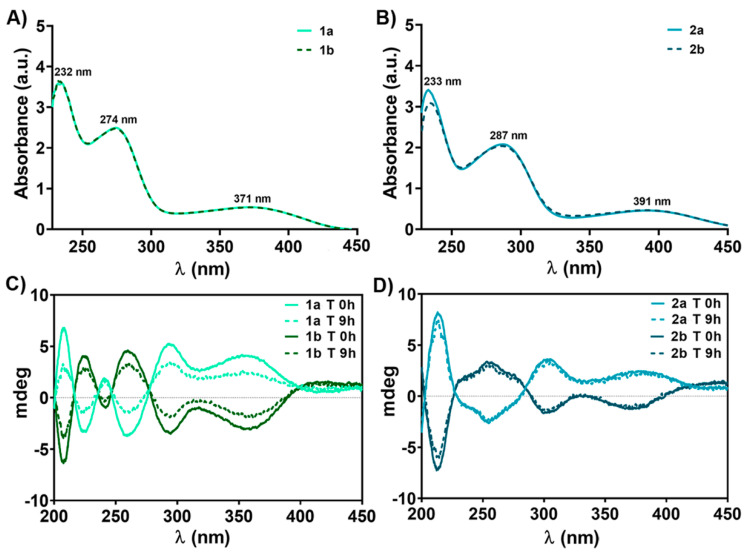
Absorption spectra (190 ÷ 450 nm) of 1 × 10^−4^ M **1a**/**1b** (**A**) and **2a**/**2b** (**B**) complexes in phosphate-buffered saline (PBS, pH 7.4) and CD spectra of 5 × 10^−4^ M **1a**/**1b** (**C**) and **2a**/**2b** (**D**) complexes in PBS, pH 7.4 outright on the prepared solutions (T 0 h) and at 9 h (T 9 h) after preparation.

The electronic spectra of oxidovanadium(V) complexes are depicted in Figure 2A,B, while those of the precursors (valine, salicylaldehyde, *o*-vanillin, V^IV^OSO_4_•3H_2_O) are presented in Supplementary Figure S1. The absorption spectra of the four oxidovanadium(V) complexes display 3 main bands in PBS (pH 7.4) solution within the studied range (Figure 2A,B). The vanadium-based compounds formation is confirmed both by the hypsochromic (blue) shift of about 52 ÷ 61 nm between the λ_max_ of benzaldehyde derivative precursors (λ_max_ = 326 nm for salicylaldehyde and λ_max_ = 348 nm of *o*-vanillin, respectively) and the second band of the complexes (274 nm for **1a**/**1b** and 287 nm for **2a**/**2b**) and by the appearance of a third band at λ_max_ = 371 nm for **1a**/**1b** and λ_max_ = 391 nm for **2a**/**2b**, which can be assigned to the charge transfer (CT) from ligand to metal (LMCT), namely from double bond oxygen (V = O) to the vanadium atom (O → V) in both **1a**/**1b** (V^V^, d^0^) and **2a**/**2b** (V^V^, d^0^) (Figure 2A,B and Supplementary Figure S1A,B). Similar to the solutions of many other vanadate(V) species and vanadium(V) bound to oxygen donor ligands, our complexes give a yellow color in PBS (pH 7.4) solution, which is primarily due to the intense LMCT bands tailing from the UV region [35].

The CD spectra of the 5 × 10^−4^ Μ vanadium compounds solutions and 2 × 10^−2^ M L-/D-valine (PBS, pH 7.4), recorded between the 200 ÷ 500 nm range, are presented in Figure 2C,D, and Supplementary Figure S2. CD spectra for oxidovanadium(V) complexes reveal the same number of bands as the corresponding UV-Vis spectra in the 230 ÷ 450 nm range (Figure 2A,B) and are in good agreement with the findings of X-ray crystallographic data (see below).

A slight deviation of λ_max_ was observed between the CD spectra of pair enantiomers and their corresponding electronic spectra (Figure 2). This observation may be due to the presence of π-π* transition of the imino (-C = N-) group [33], from the Schiff base ligand derived from salicylaldehyde and *o*-vanillin and the overlapping of n-π* and π-π* transitions, specific of precursors (λ_max_ = 252 nm and 326 nm for salicylaldehyde and 264 nm and 348 nm for *o*-vanillin, respectively) with CT from double bond oxygen (V = O) to the vanadium atom (O → V) in both **1a**/**1b** and **2a**/**2b** (Figure 2A,B, Supplementary Figure S1A,B). The similarities of the four complexes (**1a**/**1b** and **2a**/**2b**) absorption spectra suggest the presence of the same predominant conformation in solution (Figure 2). Moreover, CD spectra confirm the synthesis of both enantiomers for each compound (Figure 2C,D), and recording the spectrum of each enantiomer at the time of preparation as well as after 9 h was aimed to highlight the stability of the compounds in solution at the physiological pH and 37 °C, which will be discussed below.

### 3.3. X-Ray Crystallographic Analysis

The X-ray crystal structures of **1b** [13] and **2b** [12] have already been reported and discussed. The crystallographic data and experimental details associated with the newly synthesized compounds, **1a** and **2a**, are detailed in Supplementary Table S3.

#### 3.3.1. Description of the **1a** Structure

**Figure 3:**
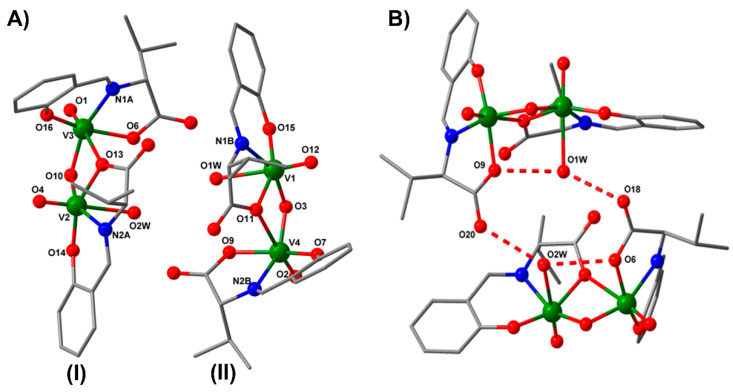
The asymmetric unit with the atom-labeling scheme of **1a**. The two independent molecules (I and II) in the asymmetric unit (**A**) and the hydrogen-bonding scheme between the carboxyl oxygens and the water molecules of **1a** (**B**). Hydrogen atoms have been excluded for clarity.

The crystallographic investigation of compound **1a** reveals the presence of a homobinuclear compound that crystalizes in the P61 chiral space group. The asymmetric unit for **1a**, presented in Figure 3A, contains two crystallographically independent molecules (I and II).

The selected bond distances and angles for **1a** are shown in Table 2. The metal ions are double bridged by one µ_2_-oxo oxygen atom and by an O atom from the carboxylate group of the ligand (monodentate bridging mode). The vanadium(V) atom shows a coordination number of 6, with two different octahedral geometry. The equatorial plane for V1 or V2 is formed by the tridentate ligand and one oxygen atom from the oxo bridge, with metal-donor atom distances varying between 1.814(5) and 2.100(6) Å for V1 and between 1.796(7) and 2.105(6) Å for V2. The apical positions are occupied by the oxygen atoms arising from the water molecules and V = O bond (V1–O1W = 2.351(7) Å, V1–O12 = 1.589(5) Å, V2–O2W = 2.386(8) Å, V2–O4 = 1.584(8) Å). The second octahedral environment for V3 and V4 is obtained by the coordination in the apical positions of two oxygen atoms from the carboxylate bridging ligand and from the V=O bond (V4–O11 = 2.413(5) Å, V4–O2 = 1.572(6) Å, V3–O1 = 1.581(7) Å, V3–O13 = 2.470(6) Å). The aqua ligands are involved in two types of H-bond interactions: (i) Intramolecular hydrogen bonds between one water molecule and one oxygen atom from coordinated carboxylate ligand (O1W–O9 = 2.766 Å, O2W–O6 = 2.839 Å); (ii) intermolecular hydrogen bonds between waters molecules and the non-coordinated carboxylate oxygen atom (O1W–O18 = 2.824 Å, O2W–O20 = 2.873 Å), resulting in supramolecular tetramers (Figure 3B).

#### 3.3.2. Description of the **2a** Structure

**Figure 4:**
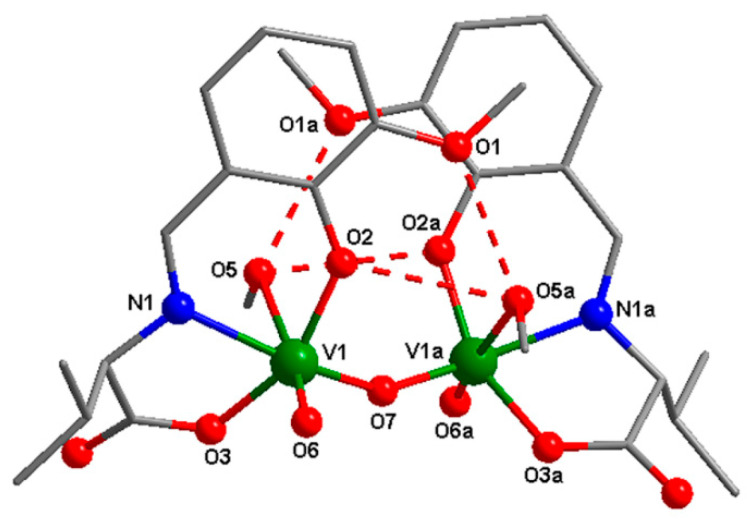
The molecular structure with the atom-labeling scheme of **2a**. For clarity, hydrogen atoms have been excluded from the diagram.

The crystal structure of compound **2a** consists of neutral oxo binuclear species (Figure 4). The selected bond distances and angles for the **2a** complex are shown in Table 3. The asymmetric units contain one vanadium(V) atom in a distorted octahedral geometry. The metal ions are coordinated by three donor atoms arising from the Schiff base ligand, one terminal oxygen atom, one oxygen from the oxo bridge, and one oxygen atom from the methanol molecule, with distances varying between 1.593(10) and 2.379(12) Å. The coordinated methanol molecule is involved in an intermolecular hydrogen bond with two oxygen atoms from the methoxy and phenoxo group (O5–O**1a** = 2.792 Å, O5–O**2a** = 2.929 Å, ^a^ = 1 − *x, y, −z* + 1).

### 3.4. Evaluation of Oxidovanadium(V) Complexes Solution Stability Over Time

In this paper, we propose to study the stability of the four oxidovanadium(V) complexes in PBS solution, at physiological pH (7.4) and temperature (37 °C).

The four vanadium complexes are soluble in methanol and DMSO. The use of methanol in medicine is forbidden since this solvent is toxic for living organisms. On the other hand, at low concentrations, DMSO performs as a good transfer substance across biological membranes [36], thus the oxidovanadium(V) compounds were dissolved in DMSO (2 × 10^−1^ M stock solutions).

**Figure 5:**
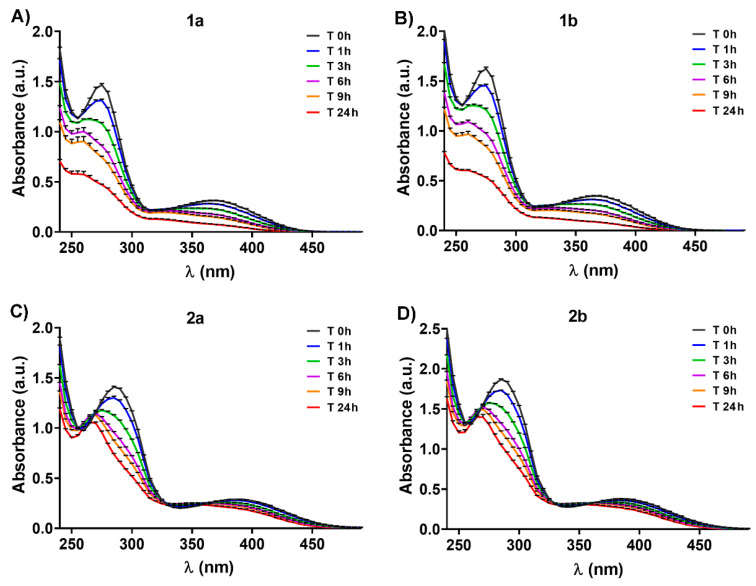
Absorption spectra (in the 230 ÷ 450 nm range) of 2 × 10^−4^ M **1a** (**A**), **1b** (**B**), **2a** (**C**), and **2b** (**D**) in phosphate-buffered saline (PBS, pH 7.4, 37 °C) recorded over 24 h.

The stability of 2 × 10^−4^ M oxidovanadium complexes in PBS at physiological pH and 37 °C was monitored by UV-Vis spectrophotometry, in the 230 ÷ 450 nm range, over 24 h (Figure 5). No detectable changes in the color (yellowish) at 37 °C were observed. The oxidovanadium(V) complexes are decomposed in a time-dependent manner when they are incubated in PBS at physiological pH and temperature, indicated by a decrease in the intensity of specific λ_max_ (Figure 5). The λ_max_ values of all complexes are shifted to the blue region with about 5 ÷ 15 nm, from T 0 h to T 24 h, an indication of a decomposition of the starting vanadium compounds, probably as a consequence of Schiff base hydrolysis [8,37] (Figure 5).

For all complexes, decreases in the intensity for both depicted λ_max_ were recorded, at 1 h (about 10% for **1a**/**1b**, and less than 10% for **2a**/**2b**, *p* < 0.05); 3 h (about 25% for **1a**/**1b**, and less than 20% for **2a**/**2b**, *p* < 0.01); 6 h (about 40% for **1a**/**1b**, and 20% for **2a**/**2b**, *p* < 0.01), 9 h (about 50% for **1a**/**1b** and 30% for **2a**/**2b**, *p* < 0.001); and 24 h (about 70% for **1a**/**1b** and 30% for **2a**/**2b**, *p* < 0.001) after solution preparation compared to initial recordings (T 0h) (Figure 5). Thus, the **2a**/**2b** enantiomers seem to be more stable at 37 °C and physiological pH than **1a**/**1b**. The existence of the isosbestic points in **2a**/**2b** spectra (Figure 5C,D) is a consequence of at least two species present in solutions. Moreover, a lower decrease in the intensity of the CD spectrum bands for all oxidovanadium(V) complexes was observed after 9 h, possibly due to the higher concentration of compound used in the CD analysis (5 × 10^−4^ M) compared to UV-Vis (2 × 10^−4^ M), which may improve their stability in PBS (Figure 2C,D). Previous studies reported that other vanadium Schiff base ligand complexes are stable in water at native pH, and only 30% of the initial quantity tested was found at pH 2 after 90 min [36,38]. Herein we evidenced for the first time that the newly developed vanadium-based compounds are stable in PBS for up to three hours, the stability decreasing in time after that. The stability of some Schiff base vanadium(V) catecholate complexes in organic or aqueous solutions was found to be greater than in biological media (for example, culture cell media), without affecting the biological functions [8,9,37]. These earlier observations may indicate that our four synthesized oxidovanadium(V) compounds exhibit the same behavior in biological environments. However, the different ways in which vanadium compounds are uptaken by cells (active or passive transport) [4] minimizes the disadvantage produced by the low stability of these compounds in biological environments, without affecting the expected biological properties [8,9,37]. Furthermore, the interaction of vanadium complexes with transporting plasma proteins assures rapid tissue delivery [39].

### 3.5. Evaluation of Oxidovanadium(V) Complexes Capacity to Bind Serum Albumin

**Figure 6:**
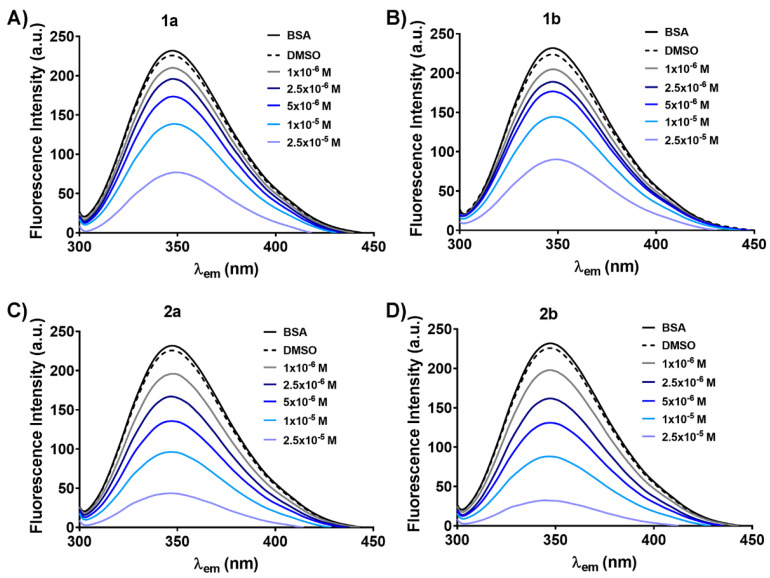
Fluorescence spectra of 2 × 10^−6^ M bovine serum albumin (BSA) in phosphate-buffered saline (PBS, pH 7.4) in the presence of various concentrations (1 ÷ 25 × 10^−6^ M) of **1a** (**A**), **1b** (**B**), **2a** (**C**), **2b** (**D**). 0.025% Dimethyl sulfoxide (DMSO, vehicle) was used as a negative control for oxidovanadium(V) complexes.

Albumin, the most abundant serum protein, is functioning as a carrier for many biological molecules and also for various drugs [39]. Thus, albumin greatly enhances the pharmacological action of different therapies [39]. Bovine serum albumin (BSA) has been extensively used as a protein model for interaction studies based on its water-soluble nature and its intrinsic fluorescence, mainly due to the two aromatic tryptophan (Trp 134 and 212) residues [23]. The fluorescence quenching method was used to study the binding capacity of vanadium compounds to BSA by showing the effect of the vanadium complexes and V^IV^OSO_4_•3H_2_O on BSA fluorescence intensity (Figure 6 and Supplementary Figure S3).

The fluorescence spectra of 2 × 10^−6^ M free BSA in PBS in the absence and the presence of 0.025% DMSO were used as controls and are shown in each graph (Figure 6 and Supplementary Figure S3). When excited at 295 nm, BSA in PBS solution (pH 7.4) exhibits a strong fluorescence emission with a maximum λ_em_ at 347 nm due to the Trp residues [40]. The oxidovanadium(V) complexes diminish the fluorescence intensity of BSA in a dose-dependent manner without the modification of the maximum emission wavelength (Figure 6). The decrease was about 60% for **1a**/**1b** and 80% for **2a**/**2b** for the highest tested concentration compared to free BSA and BSA with DMSO (Figure 6). Interestingly, the vanadium ion precursor, V^IV^OSO_4_•3H_2_O, does not reduce the fluorescence intensity of BSA even at the highest concentration used (Supplementary Figure S3). This result may be a consequence of vanadium oxidation state in complexes (V^V^) compared to V^IV^ in V^IV^OSO_4_•3H_2_O of which exposure to PBS at pH 7.4 avoids its oxidation to vanadium(V) known to be essential for ligands dissociation and replacement with stronger ligands from biological media [21,41,42]. The oxidation state of oxidovanadium(V) compounds may favor the Schiff base ligands replacement and rapid coordination of amino acid residues from the albumin active site to vanadium(V).

To evaluate the binding mechanism of vanadium complexes to BSA, we used the Stern–Volmer equation [23] to fit the data as described in the Supplementary material. The values of the Stern-Volmer constant, obtained from the slope of the linear plot (Supplementary Figure S4) and the collision quenching constant (K_q_ = K_sv_/τ_0_) for vanadium complexes are depicted in Table 4. The binding of quenchers to the biopolymers can be explained through two different mechanisms: Dynamic—characterized by a maximal K_q_ of 2.0 × 10^10^ M^−1^s^−1^ (for fluorescence lifetime of 10^−8^ s) and static—indicated by higher values of K_q_ [12,23]. The K_q_ for each vanadium-based complex is higher than K_q_ = 2.0 × 10^10^ M^−1^s^−1^, which strongly indicates that the fluorescence quenching of BSA is caused mainly by a specific static interaction [43]. Moreover, the complexes **2a** and **2b** reduce the fluorescence intensity of BSA 2 times and 4 times compared to **1a** and **1b**, respectively (Table 4). Therefore, it can be estimated that **2a** and **2b** bind firmly to BSA than **1a** and **1b**, and this may be a consequence of establishing additional interactions (for example hydrogen bonds) between the O-CH_3_ groups of **2a**/**2b** complexes and amino acid residues from the active site of BSA [44]. Interestingly, a slight difference between S and R-enantiomers can be observed. **1a** decreases the BSA fluorescence intensity by 1.34 times compared to **1b**, while **2a** has a lower quenching capacity (by 0.69 times versus **2b**) (Table 4), probably as a consequence of different structural orientation of each enantiomer during the interactions with Trp residues of BSA.

### 3.6. Anti-Diabetic Activity of Oxidovanadium(V) Complexes

Previous reports show that the vanadium salts can induce the activation of many components of the insulin signaling pathway including the insulin receptor and downstream proteins [16,45,46]. Despite their promising anti-diabetic effects, the vanadium salts exhibit low absorption and bioavailability and are cytotoxic at high administered doses [47,48]. Recent evidence suggests that coordinating different biological ligands to vanadium centers has improved the compound’s stability, absorption, and bioavailability, reduced its toxicity, and enhanced its biological properties [48,49,50].

**Figure 7:**
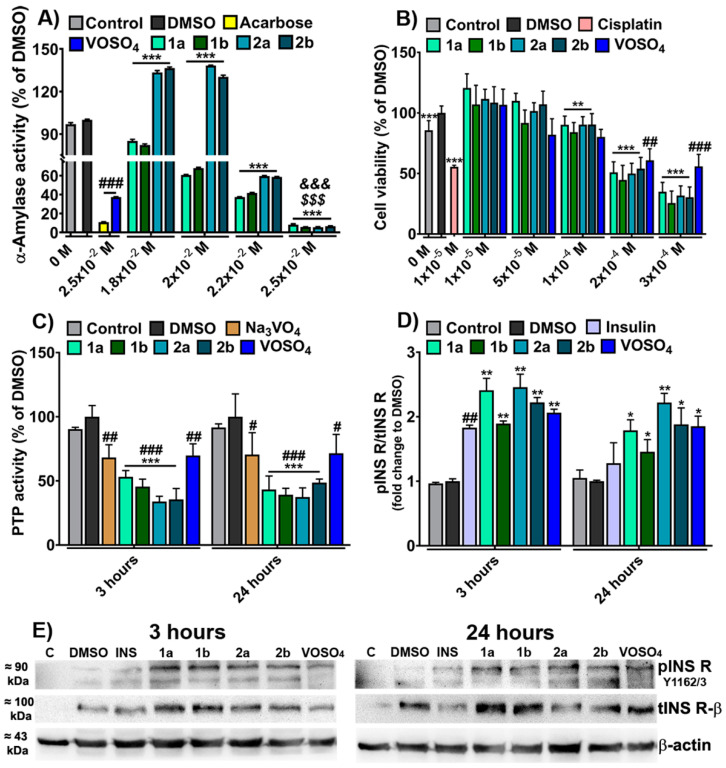
The effect of **1a**, **1b**, **2a**, **2b** on α-amylase activity (**A**), HepG2 cell viability (**B**), total protein tyrosine phosphatases (PTP) enzymatic activity (**C**), insulin receptor (INS R) phosphorylation (**D**), and representative immunoblotting images of the phosphorylated form of INS R (pINS R), total form of INS R (tINS R-β), and β-actin (**E**). Acarbose was used as a positive control for α-amylase inhibition. Cisplatin was used as a positive control for cytotoxicity, Na_3_V^V^O_4_ was used as a control for PTP inhibition. As a negative control for V^IV^OSO_4_•3H_2_O, Na_3_V^V^O_4_ (VOSO_4_ and Na_3_VO_4_ for chart simplification), and insulin treatment, HepG2 cells exposed to the free-complete medium were used (depicted barely control). Dimethyl sulfoxide (DMSO) was used as a negative control for oxidovanadium(V) complexes and cisplatin treatment. The results were expressed as % of DMSO and were showed as mean ± SD and analyzed using unpaired two-tailed Student’s *t*-test; ^*^ *p* < 0.05, ^**^ *p* < 0.01, ^***^ *p* < 0.001 vs. DMSO and **^#^** *p* < 0.05, **^##^** *p* < 0.01, **^###^** *p* < 0.001 vs. Control, **^&&&^** *p* < 0.001 vs. acarbose and **^$$$^** *p* < 0.001 vs. V^IV^OSO_4_•3H_2_O.

Our data fulfill these observations, showing that the newly developed compounds **1a** and **2a** and their S-enantiomers **1b** and **2b** are stable in PBS at physiological pH and temperature for up to three hours and possess albumin binding properties (Figure 5 and Figure 6). Based on these encouraging results, the potential anti-diabetic properties of the newly developed compounds **1a** and **2a** and their S-enantiomers **1b** and **2b** were investigated (Figure 7).

#### 3.6.1. In Vitro α-Amylase Inhibition Test

The initial step in glucose metabolism is completed by the salivary α-amylase that partially hydrolyzes food carbohydrates, followed by the total intestinal hydrolysis of carbohydrates by the pancreatic α-amylase [18]. To investigate the in vitro anti-diabetic activity of the synthesized vanadium-based compounds, we assessed the inhibitory effect of the oxidovanadium(V) complexes (1.8 × 10^−2^–2.5 × 10^−2^ M) on α-amylase activity (Figure 7A).

The V^IV^OSO_4_•3H_2_O (2.5 × 10^−2^ M) was used as a control for metal ions, the well-known anti-diabetic drug acarbose (2.5 × 10^−2^ M) was used as a positive control for α-amylase activity inhibition [51], and 12.5% DMSO (vehicle) was used as a negative control for complexes. As a negative control for acarbose and V^IV^OSO_4_•3H_2_O (depicted barely control in Figure 7A), a sample containing only starch and enzyme was used. The inhibitory effect of the vanadium-based complexes is associated with the reduced α-amylase activity (Figure 7A). At 1.8 × 10^−2^ and 2.0 × 10^−2^ M, **1a** and **1b** exert a significant inhibitory effect (more than 18%, *p* < 0.001), while for **2a** and **2b**, the enzyme activity is increasing with 35%, *p* < 0.001, compared to DMSO. Moreover, at 2.5 × 10^−2^ M, all four vanadium compounds exhibit a higher inhibitory effect on α-amylase activity compared to the positive control (acarbose) and V^IV^OSO_4_•3H_2_O (more than 23% and 77%, respectively, *p* < 0.01). At this concentration, the compounds reduce more than 90% of the enzyme’s activity, compared to DMSO and the control sample (*p* < 0.001) (Figure 7A). The inhibitory effect of all four complexes on α-amylase activity is increased as the compound concentration increases (Figure 7A) and this observation is consistent with their ability to function as albumin quenching agents (Figure 6).

#### 3.6.2. Cytotoxicity Assay

The cytotoxic effect of oxidovanadium(V) compounds on the HepG2 cell line was assessed by XTT assay. The viability of HepG2 cells exposed for 24 h to different concentrations of vanadium compounds (1 × 10^−5^–3 × 10^−4^ M) is depicted in Figure 7B. The IC_50_ values are summarized in Supplementary Table S4.

The vanadium complexes have none or low cytotoxic effects on HepG2 cells at concentrations ranging between 5 × 10^−5^ M and 1 × 10^−4^ M, the viability decreasing by about 7% at 1 × 10^−4^ M compared to DMSO treated cells, considered 100% viable (*p* < 0.01). The **1a** and **2a** compounds induce an increase of the cell viability at 1 × 10^−5^ M (by 11%, *p* < 0.001) compared to the cells treated with DMSO (Figure 7B). DMSO treatment also increases cell viability (about 16%, *p* < 0.001) compared to the control untreated cells (Figure 7B), a result in agreement with previous reports [52]. It can be assumed that the significant increase of cell viability after treatment with **1a** and **2a** complexes may be a consequence of both the proliferative effect of DMSO and of compounds per se since this tendency was also observed in the case of V^IV^OSO_4_•3H_2_O (24% increases, *p* < 0.001) compared to control cells (Figure 7B).

Moreover, the oxidovanadium(V) compounds induce the death of more than 46% of HepG2 cells at 2 × 10^−4^ M and above 65% at 3 × 10^−4^ M compared to DMSO treated cells (*p* < 0.001). By comparison, the vanadium precursor, V^IV^OSO_4_•3H_2_O, reduces the HepG2 viability only by 34% at the highest concentration (*p* < 0.001) compared to the control cells (Figure 7B). The IC_50_, which represents the concentration of complexes that induce a growth inhibition by 50%, is above 2 × 10^−4^ M for all compounds (Supplementary Table S4). At 1 × 10^−5^ M, the anti-tumor drug cisplatin reduces by 44% (*p* < 0.001) the viability of the HepG2 cells compared to DMSO treated cells (Figure 7B), the IC_50_ value of cisplatin being 1.1 × 10^−5^ M (Supplementary Table S4).

The data show that the IC_50_ values for all vanadium compounds are higher than the IC_50_ of cisplatin but lower than IC_50_ of V^IV^OSO_4_•3H_2_O (Figure 7B and Supplementary Table S4). Our results suggest that the four here synthesized vanadium-based compounds are cytocompatible with the tested cells and do not induce marked cytotoxicity at concentrations lower than 2 × 10^−4^ M. For this reason, we further set out to evaluate their in vitro potential to mimic the biological effects of the hormone insulin and their anti-diabetic action.

#### 3.6.3. Quantification of Total PTP Enzymatic Activity and Insulin Receptor Phosphorylation

A well-documented mechanism by which vanadium-based compounds sensitize the insulin signaling pathway comprises the inhibition of the activity of different PTPs as a result of the vanadate-phosphate antagonism [2,21]. The PTP inhibitory activity of the four synthesized vanadium-based compounds and the ability to mimic the effect of insulin is shown in Figure 7C,D.

The HepG2 cells were treated with a non-cytotoxic concentration (2.5 × 10^−5^ M) of synthesized oxidovanadium(V) compounds for 3 and 24 h to establish their anti-diabetic activity and the capacity to mimic the effect of the insulin hormone. We show that all four vanadium-based compounds significantly inhibit (more than 45%, *p* < 0.001) the intracellular total PTP activity compared to cells treated with the vehicle (DMSO) and to control cells (untreated) (Figure 7C). Moreover, the oxidovanadium(V) compounds reduce the PTP activity by more than 20% (*p* < 0.01) at 3 h and 24 h compared to Na_3_V^V^O_4_ and V^IV^OSO_4_•3H_2_O (Figure 7C). This result points out that the inhibitory activity of vanadium-based compounds is higher than that of the well-known PTP inhibitor, Na_3_V^V^O_4_, and of their vanadium precursor.

The PTP inhibitory effects of the oxidovanadium(V) complexes are consistent with the immunoblotting quantification of the phosphorylated form of insulin receptor (pINS R) (Figure 7D,E). Three hours after cell treatment, all four synthesized compounds and their precursor highly phosphorylate (with more than 100%, *p* < 0.01) the INS R, similar to the insulin hormone, which increases the INS R phosphorylation by about 80% after treatment compared to its negative control (cells exposed to free-complete medium) (*p* < 0.01) (Figure 7D,E). After 24 h of cell treatment, the action of insulin is reduced and insignificant compared to control cells, while the pINS R levels are maintained higher (more than 40%, *p* < 0.05) when the cells were treated with the four oxidovanadium(V) compounds and their precursor compared to cells exposed to vehicle (DMSO) and untreated cells (control).

This observation reveals for the first time that the herein reported vanadium-based compounds keep the insulin receptor phosphorylated for a long time compared to the insulin hormone. These data correlate with the increased ability of these compounds to inhibit total PTP activity. Our results are in good agreement with previous in vitro and in vivo studies that reveal the insulin sensitization properties of bis(maltolato)oxidovanadium(IV) (BMOV) and other vanadium-based compounds, which comprises PTP inhibition [14,22] followed by insulin receptor phosphorylation [20,21,53].

## 4. Conclusions

Two new binuclear oxidovanadium(V) complexes (**1a** and **2a**) and their previously reported S-enantiomers (**1b** and **2b**) were synthesized and characterized in the present study. The structures of **1a** and **2a** were determined by X-ray crystallography and UV-Vis/ IR spectral analysis. The enantiomeric relationship between *R* and *S* isomers was established by circular dichroism studies and was supported by X-ray crystallography. All four complexes exhibit stability in solution at physiological pH and 37 °C for up to three hours and a moderate to high rate of decomposition after this period, which may suggest that the newly developed oxidovanadium(V) complexes are decomposed in the biological media, most likely as a consequence of Schiff base hydrolysis. The fluorimetry assay showed that all complexes have a dose-dependent binding capacity to Trp residues of BSA in a specific static interaction. Moreover, the **2a**/**2b** bind firmly to BSA compared to **1a**/**1b**, which may be a consequence of establishing additional interactions between the O-CH_3_ groups of **2a**/**2b** and amino acids from the active site of albumin. All four oxidovanadium(V) complexes have in vitro anti-diabetic activities and insulin-mimetic effects, revealed by the reduction of α-amylase and total PTP activity and increasing the phosphorylation of INS R in HepG2 cells.

To the best of our knowledge, this is the first study reporting the insulin-mimetic and anti-diabetic properties of the two newly synthesized oxidovanadium(V) compounds (**1a** and **2a**) and their S-enantiomers (**1b** and **2b**). Our compounds inhibit α-amylase and total PTP activity and sensitize the insulin pathway, features that correlate with compounds’ stability at physiological pH and temperature and with their higher capacity to bind serum albumin. Additionally, the vanadium-based complexes exhibit none or low cytotoxic effects on HepG2 cells, at concentrations below 2 × 10^−4^ M, the IC_50_ of these compounds being higher than that observed for the antineoplastic agent cisplatin.

Based on the properties described above, we conclude that the two new synthesized oxidovanadium(V) complexes and their known S-enantiomers may be promising candidates for further in vitro and in vivo biological analysis to introduce them as drugs for the treatment of patients with type 2 diabetes.

## Figures and Tables

**Table 1:** Electronic spectral data of 1 × 10^−4^ M **1a**, **1b**, **2a**, and **2b** complexes in PBS, pH 7.4.

Compound	λ_max_ (nm)	ε (M^−1^ cm^−1^)	Assignments
**1a**	232 274 371	35796 24957 5432	1st band (232, 233 nm): π → π* transition of the benzene ring and charge-transfer transitions [13], 2nd band (274, 287 nm): the π → π* transitions of the benzene ring [33] and to imino (-CH = N-) group coordination [34], 3rd band (371, 391 nm): probably due to the O → V CT ^1^ from double bond oxygen to the vanadium atom [35].
**1b**	232 274 371	36574 24733 5384	
**2a**	233 287 391	34090 20811 4586	
**2b**	233 287 391	30692 20355 4659	

^1^ CT = charge transfer.

**Table 2:** Selected bond lengths (Å) and angles (**^o^**) for **1a**.

Bond	(Å)	Angle	(^o^)	Angle	(^o^)
V1—O12	1.589(5)	O12—V1—O3	101.4(3)	O4—V2—O14	101.9(5)
V1—O3	1.814(5)	O12—V1—O15	100.8(3)	O4—V2—O10	102.4(4)
V1—O15	1.824(6)	O3—V1—O15	108.4(3)	O14—V2—O10	105.7(3)
V1—O11	1.960(5)	O12—V1—O11	99.3(3)	O4—V2—O13	98.8(4)
V1—N1B	2.100(6)	O3—V1—O11	82.2(2)	O14—V2—O13	154.8(3)
V1—O1W	2.351(7)	O15—V1—O11	154.8(3)	O10—V2—O13	83.5(2)
V4—O2	1.572(6)	O12—V1—N1B	95.6(3)	O4—V2—N2A	96.5(3)
V4—O3	1.797(6)	O3—V1—N1B	154.8(2)	O14—V2—N2A	86.4(3)
V4—O7	1.844(6)	O15—V1—N1B	86.2(2)	O10—V2—N2A	154.8(3)
V4—O9	1.948(6)	O11—V1—N1B	76.8(2)	O13—V2—N2A	77.1(3)
V4—N2B	2.110(7)	O12—V1—O1W	175.2(3)	O4—V2—O2W	176.4(3)
V4—O11	2.413(5)	O3—V1—O1W	83.1(2)	O14—V2—O2W	78.7(3)
		O15—V1—O1W	79.3(3)	O10—V2—O2W	80.9(3)
V1—V4	3.088(18)	O11—V1—O1W	79.4(2)	O13—V2—O2W	79.8(3)
		N1B—V1—O1W	79.6(2)	N2A—V2—O2W	79.9(3)
V2—O4	1.584(8)	V4—O3—V1	117.5(3)	V3—O10—V2	117.9(3)
V2—O14	1.796(7)	V1—O11—V4	89.23(19)	V2—O13—V3	87.4(2)
V2—O10	1.811(6)	O2—V4—O3	103.9(3)	O1—V3—O10	103.7(3)
V2—O13	1.955(5)	O2—V4—O7	99.1(3)	O1—V3—O16	99.6(4)
V2—N2A	2.105(7)	O3—V4—O7	99.6(2)	O10—V3—O16	99.1(3)
V2—O2W	2.386(8)	O2—V4—O9	98.3(3)	O1—V3—O6	98.3(4)
V3—O1	1.581(7)	O3—V4—O9	91.5(3)	O10—V3—O6	92.8(3)
V3—O10	1.784(6)	O7—V4—O9	156.5(3)	O16—V3—O6	155.5(3)
V3—O16	1.833(8)	O2—V4—N2B	104.2(3)	O1—V3—N1A	104.5(3)
V3—O6	1.959(7)	O3—V4—N2B	150.7(2)	O10—V3—N1A	150.7(3)
V3—N1a	2.096(7)	O7—V4—N2B	84.3(3)	O16—V3—N1A	83.7(3)
V3—O13	2.470(6)	O9—V4—N2B	76.2(3)	O6—V3—N1A	75.7(3)
		O2—V4—O11	174.5(3)	O1—V3—O13	173.9(3)
V2—V3	3.080(2)	O3—V4—O11	70.6(2)	O10—V3—O13	70.3(2)
		O7—V4—O11	82.6(2)	O16—V3—O13	82.8(3)
		O9—V4—O11	81.6(2)	O6—V3—O13	81.2(2)
		N2B—V4—O11	81.2(2)	N1a—V3—O1	81.3(3)

**Table 3:** Selected bond lengths (Å) and angles (**^o^**) for **2a**.

Bond	(Å)	Angle	(^o^)	Angle	(^o^)
O5—V1 O6—V1 N1—V1 O2—V1 O3—V1 O7—V1 O7—V1 ^a#^ O5—V1 ^a#^ V1…V1 ^a^	2.379(12) 1.593(10) 2.137(13) 1.869(12) 1.924(11) 1.797(5) 1.797(5) 2.790(184) 3.473(3)	O6—V1—O7 ^a#^ O6—V1—O2 O7—V1—O2 O6—V1—O3 O7—V1—O3 O2—V1—O3 O6—V1—N1 O7—V1—N1	102.3(5) 101.6(5) 95.1(6) 96.8(5) 98.0(5) 154.5(5) 97.0(5) 160.4(4)	O2—V1—N1 O3—V1—N1 O6—V1—O5 O7—V1—O5 O2—V1—O5 O3—V1—O5 N1—V1—O5 V1-O7-V1 ^a^	84.4(5) 76.0(5) 173.3(5) 83.9(4) 80.2(5) 79.7(5) 76.7(4) 150.2

^a#^ = 1: −x, y, −z + 1.

**Table 4:** The Stern–Volmer constant (K_SV_) and collision quenching constant (K_q_) of the Stern–Volmer equation for **1a**, **1b**, **2a**, and **2b** complexes.

Compound	K_SV_ (M^−1^)	K_q_ (M^−1^s^−1^)	K_q(2a)_/K_q(1a)_	K_q(2b)_/K_q(1b)_	K_q(1a)_/K_q(1b)_	K_q(2a)_/K_q(2b)_
** 1a **	0.807 × 10^5^	8.07 × 10^12^	2.12	4.14	1.34	0.690
** 1b **	0.601 × 10^5^	6.01 × 10^12^				
** 2a **	1.718 × 10^5^	1.718 × 10^13^				
** 2b **	2.489 × 10^5^	2.489 × 10^13^				

## Data Availability

All data supporting reported results are included in the article.

## Supplementary Materials

The following are available online at https://www.mdpi.com/article/10.3390/biomedicines9050562/s1, Figure S1: Absorption spectra of 5 × 10^−4^ M salicylaldehyde (A), *o*-vanillin (B), 2 × 10^−3^ M D-/L-valine (C), and 7.2 × 10^−2^ M V^IV^OSO_4_●3H_2_O (D) in phosphate-buffered saline (PBS) at pH 7.4, Figure S2: CD spectra of 2 × 10^−2^ M L-/D-valine in phosphate-buffered saline (PBS) at pH 7.4, Figure S3: Fluorescence spectra of the 2 × 10^−6^ M BSA in phosphate-buffered saline (PBS) at pH 7.4, in the presence of various concentrations (1–25 × 10^−6^ M) of V^IV^OSO_4_●3H_2_O, Figure S4: The plots of I_0_/I vs. [Q] for **1a** (A), **1b** (B), **2a** (C), and **2b** (D), Table S1: The main infrared absorption frequencies (cm^−1^) corresponding to various groups for **1a**, **1b**, **2a**, and **2b** complexes, Table S2: The main infrared absorption frequencies (cm^−1^) corresponding to various groups in the precursors of oxidovanadium(V) complexes, Table S3: The crystallographic data of **1a** and **2a**, Table S4: Summary of IC_50_ values (M) calculated for **1a**, **1b**, **2a**, **2b**, V^IV^OSO_4_●3H_2_O, and cisplatin from dose-response cytotoxicity data generated by XTT assay, measuring the viability of HepG2 cells exposed for 24 h to the compounds.
