# Supplementary material for: Synthesis, Characterization, and In Vitro Insulin-Mimetic Activity Evaluation of Valine Schiff Base Coordination Compounds of Oxidovanadium(V)
Source: Biomedicines. 2021 May 17;9(5):562. doi: 10.3390/biomedicines9050562 (PMC8156053; doi:10.3390/biomedicines9050562)
Supplement: Supplementary file 1 [file biomedicines-09-00562-s001.zip › biomedicines-1203169-supplementary.pdf]

Supplementary material

Synthesis, Characterization, and In Vitro Insulin-Mimetic Activity Evaluation of Valine Schiff Base Coordination Compounds of Oxidovanadium(V)

Mihaela Turtoi[1*], Maria Anghelache[1], Andrei A. Patrascu[2], Catalin Maxim[2], Ileana Manduteanu[1], Manuela Calin[1*], Delia-Laura Popescu[2*]

[1] ”Medical and Pharmaceutical Bionanotechnologies” Laboratory, Institute of Cellular Biology and Pathology ”Nicolae Simionescu” of the Romanian Academy, 8 B.P. Hasdeu, 050568-Bucharest, Romania; mihaela.carnuta@icbp.ro, maria.anghelache@icbp.ro, ileana.manduteanu@icbp.ro, manuela.calin@icbp.ro.

[2] Department of Inorganic Chemistry, Faculty of Chemistry, University of Bucharest, 23 Dumbrava Roşie, 020464-Bucharest, Romania; andrei_alunel@yahoo.com, catalin.maxim@chimie.unibuc.ro, delia.popescu@chimie.unibuc.ro.

*Corresponding authors:

M.T. mihaela.carnuta@icbp.ro

M.C. manuela.calin@icbp.ro

D.-L.P. delia.popescu@chimie.unibuc.ro

Table of Contents

	Page
**Methods**	**3**
**The Stern–Volmer equation**	**3**
**Results**	**4**
**Figure S1**	**4**
**Figure S2**	**4**
**Figure S3**	**5**
**Figure S4**	**5**
**Table S1**	**6**
**Table S2**	**6**
**Table S3**	**6**
**Table S4**	**7**
**References**	**8**

1. Methods

The Stern–Volmer equation

The Stern–Volmer equation [1] is:

$$I_0/I = 1 + K_q\tau_0[Q] = 1 + K_{sv}[Q],$$

where: I_0 = the fluorescence intensities of free BSA solution at 347 nm,

I = the fluorescence intensities of BSA solution at 347 nm in the presence of vanadium complexes,

[Q] = the concentration of vanadium complexes,

τ_0 = the fluorescence lifetime of the biopolymer (10^{-8} s),

K_q = the collision quenching constant;

K_{sv} = the Stern-Volmer constant.

Stern–Volmer plots of the BSA fluorescence quenching in the presence of each compound are represented in Fig. S4, together with their linear best fit in which y = I_0/I, x = [Q], a = slope of the line, and b = y-intercept. The plots exhibit a good linear relationship with R^2 between 0.9794 and 0.9965. The fluorescence intensities are the average of three recordings measured at a scanning speed of 250 nm/min, with an excitation and emission bandwidth of 5 nm.

2. Results

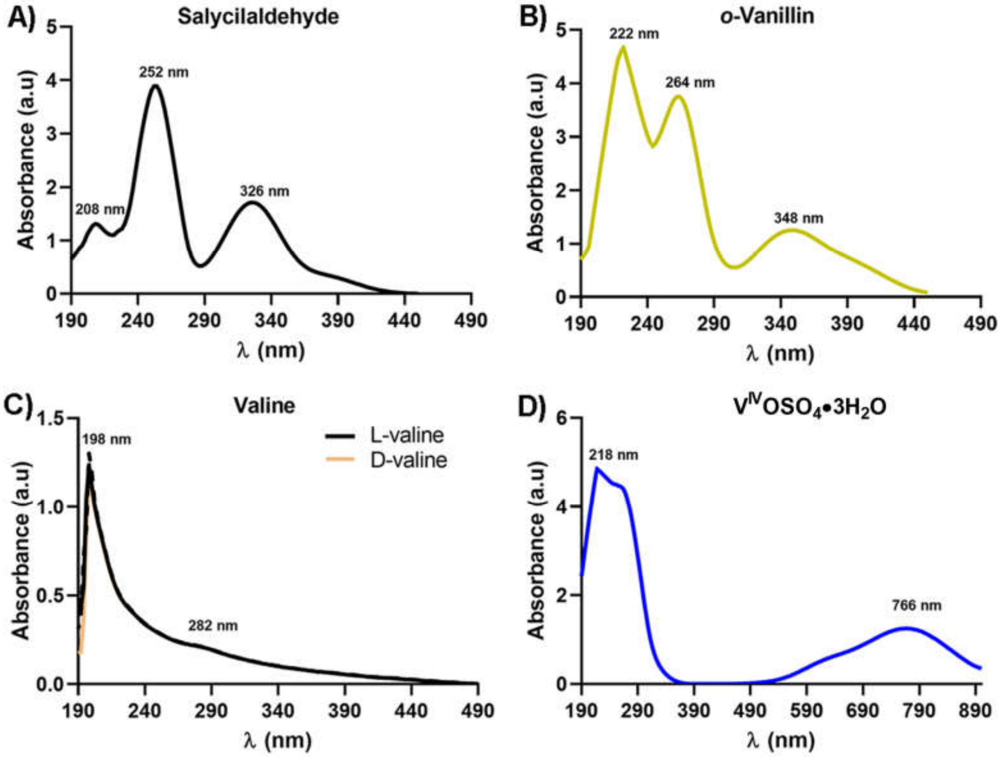

Figure S1. Absorption spectra of 5x10^{-4} M salicylaldehyde (**A**), *o*-vanillin (**B**), 2x10^{-3} M D-/L-valine (**C**), and 7.2x10^{-2} M $V^{IV}OSO_4$•$3H_2O$ (**D**) in phosphate-buffered saline (PBS) at pH 7.4.

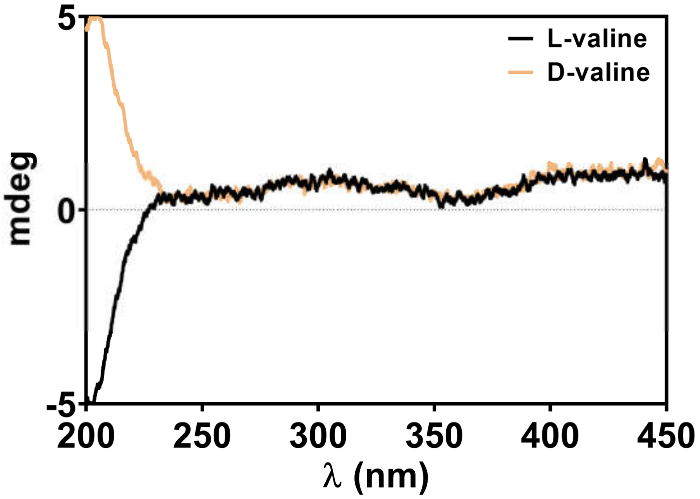

Figure S2. CD spectra of 2x10^{-2} M L-/D-valine in phosphate-buffered saline (PBS) at pH 7.4.

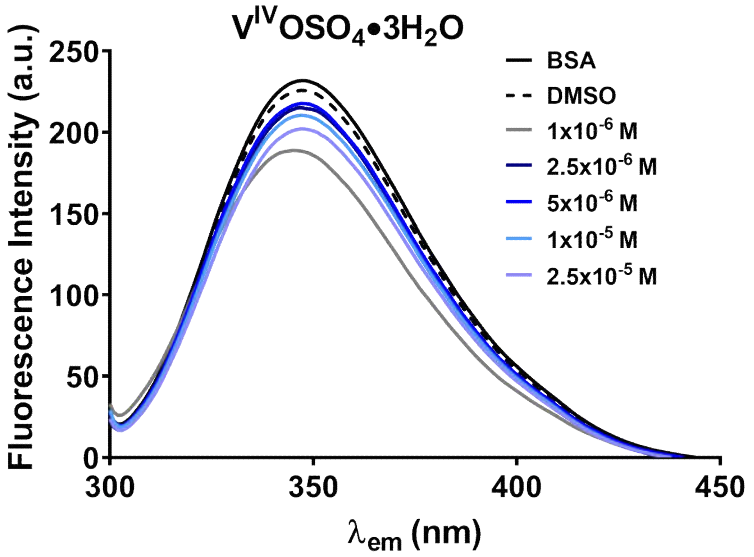

Figure S3. Fluorescence spectra of the 2x10^{-6} M BSA in phosphate-buffered saline (PBS) at pH 7.4, in the presence of various concentrations (1-25x10^{-6} M) of $V^{IV}OSO_4$•$3H_2O$.

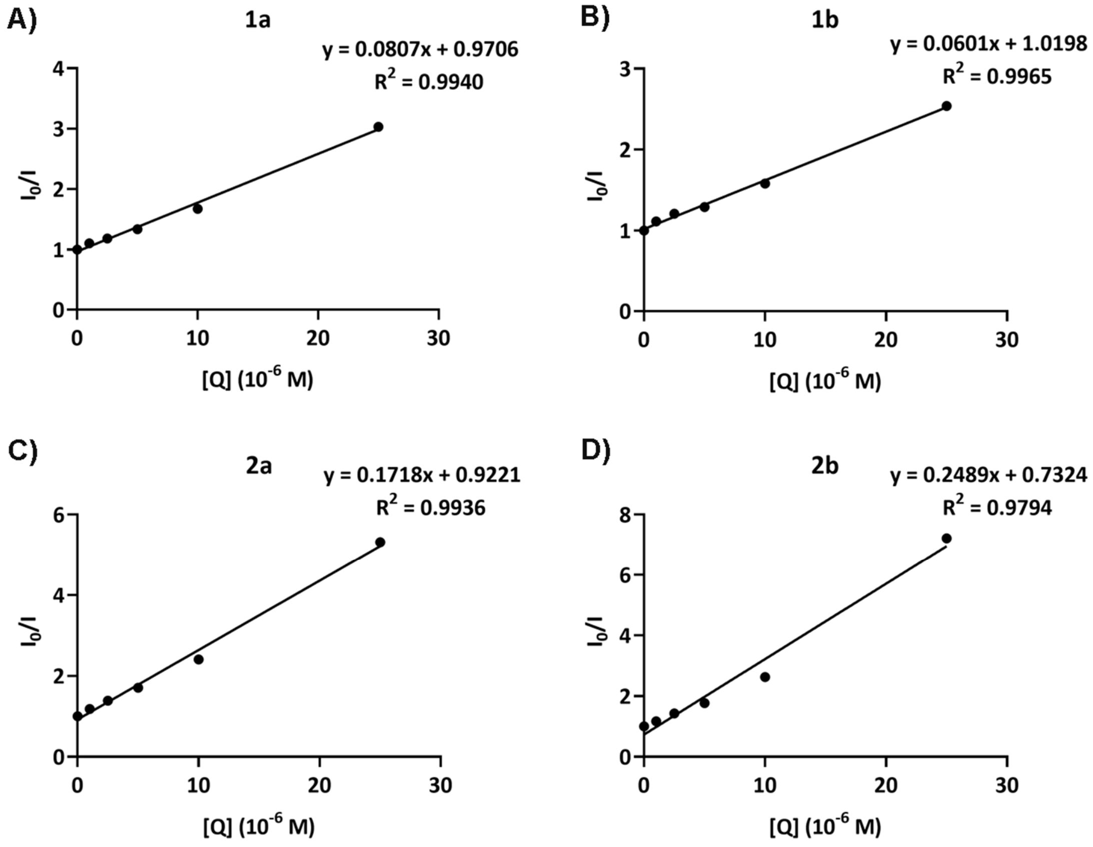

Figure S4. The plots of I_0/I *vs.* [Q] for 1a (**A**), 1b (**B**), 2a (**C**), and 2b (**D**).

Table S1. The main infrared absorption frequencies (cm^{-1}) corresponding to various groups for 1a, 1b, 2a, and 2b complexes.

ν (cm⁻¹)	1a	1b	2a	2b
νO-H	3703	3701	3702-3400	3702-3400
νC-H methyl	2966-2870	2966-2870	2966-2872	2966-2872
νC=N	1712-1683	1712-1683	1680	1680
$\nu_{as}COO^-$	1602	1602	1627	1627
νC=C	1554	1554	1570	1570
$\nu_{s}COO^-$	1394	1394	1411	1411
νC_{Ph}-O	1286	1286	1259	1259
νV=O	991	991	974	974
ν_{as}(V-O-V)	761	761	744	744
νV–N	571	571	597	597
νV–O	451	451	459	459

Table S2. The main infrared absorption frequencies (cm⁻¹) corresponding to various groups in the precursors of oxidovanadium(V) complexes.

υ (cm⁻¹)	Valine	Salicylaldehyde	*o*-Vanillin
*υ*O-H	3160	3064	3015
*υ*C-H methyl	2958	-	2973
*υ*N-H	2625-2110	-	-
*υ*C=O	-	1664	1639
$\upsilon_{as}COO^-$	1612	-	-
*υ*C=C	-	1487	1455
$\upsilon_{s}COO^-$	1323	-	-
*υ*O-H	-	1386	1389
υC_{Ph}-O	-	1276	1259

Table S3. The crystallographic data of 1a and 2a.

Compound	1a	2a
Formula	$C_{48}H_{56}N_4O_{20}V_4$	$C_{28}H_{34}N_2O_{13}V_2$
Formula weight/ g mol⁻¹	1212.72	710.47
T/K	293(2)	293(2)
λ/Å	0.71073	0.71073
Crystal system	Hexagonal	Monoclinic
Space group	$P6_1$	C2
Unit cell		
a/Å	14.5093(2)	23.399(5)
b/Å	14.5093(4)	9.4811(19)
c/Å	45.9511(4)	7.4902(15)
α/deg	90	90
β/deg	90	92.67(3)
γ/deg	120	90
V/Å³	8377.6(2)	1659.9(6)
Z	6	2
Calculated density/g cm⁻³	1.442	1.421
Absorption coefficient/cm⁻¹	0.724	0.627
F(000)	3744	736
Crystal size/mm × mm × mm	0.24 x 0.12 x 0.09	0.4 x 0.2 x 0.1
θ range/deg	1.621 to 24.508	2.318 to 24.999
Limiting indices	-16 < h < 16,<br>-16 < k < 16<br>-53 < l < 49	-27 < h < 27,<br>-10 < k < 11,<br>-8 < l < 8

Collected reflections	36236	4602
Symmetry independent reflections	9016	2453
Rint	0.0583	0.1662
Data/restraints/ parameters	9016 / 13 / 696	2453 / 1 / 204
GOF on F2	1.064	1.132
Final R indices	R1 = 0.0573<br>wR2 = 0.1502	R1 = 0.0832<br>wR2 = 0.1882
Largest diff peak and hole/e Å−3	0.546 and -0.342	0.613 and -0.515
Flack parameter	0.01(2)	0.06(13)

Table S4. Summary of IC_{50} values (M) calculated for 1a, 1b, 2a, 2b, $V^{IV}OSO_4 \bullet 3H_2O$, and cisplatin from dose-response cytotoxicity data generated by XTT assay, measuring the viability of HepG2 cells exposed for 24 hours to the compounds.

**Compound**	**IC_{50} (M) ± SD (M)**
1a	$2.3 x 10^{-4} \pm 2.4 x 10^{-5}$
1b	$2.0 x 10^{-4} \pm 4.3 x 10^{-5}$
2a	$2.3 x 10^{-4} \pm 2.2 x 10^{-5}$
2b	$2.3 x 10^{-4} \pm 2.9 x 10^{-5}$
$V^{IV}OSO_4 \bullet 3H_2O$	$3.3 x 10^{-4} \pm 15.8 x 10^{-5}$
Cisplatin	$1.1 x 10^{-5} \pm 4.9 x 10^{-7}$

References

1. Guo, Q.; Li, L.; Dong, J.; Liu, H.; Xu, T.; Li, J. Synthesis, crystal structure and interaction of l-valine Schiff base divanadium(V) complex containing a V2O3 core with DNA and BSA. *Spectrochim. Acta Part A Mol. Biomol. Spectrosc.* **2013**, *106*, 155–162, doi:10.1016/j.saa.2012.12.089.
